# Telemedicine and the Use of Korean Medicine for Patients With COVID-19 in South Korea: Observational Study

**DOI:** 10.2196/20236

**Published:** 2021-01-19

**Authors:** Soobin Jang, Dongsu Kim, Eunhee Yi, Gunhee Choi, Mideok Song, Eun-Kyoung Lee

**Affiliations:** 1 Clinical Medicine Division Korea Institute of Oriental Medicine Daejeon Republic of Korea; 2 KM Policy Research Center Korea Institute of Oriental Medicine Daejeon Republic of Korea; 3 College of Oriental Medicine Dongshin University Jeollanam-do Republic of Korea; 4 Medical/Information and Communications Affairs The Association of Korean Medicine Seoul Republic of Korea; 5 Department of Academic Affairs The Association of Korean Medicine Seoul Republic of Korea; 6 Research Institute of Korean Medicine Policy The Association of Korean Medicine Seoul Republic of Korea

**Keywords:** telemedicine, telehealth, herbal medicine, Korean medicine, COVID-19, Korea, pandemic, guideline, infectious disease

## Abstract

**Background:**

COVID-19 was first reported in Wuhan, China, in December 2019, and it has since spread worldwide. The Association of Korean Medicine (AKOM) established the COVID-19 telemedicine center of Korean medicine (KM telemedicine center) in Daegu and Seoul.

**Objective:**

The aim of this study was to describe the results of the KM telemedicine center and the clinical possibility of using herbal medicines for COVID-19.

**Methods:**

All procedures were conducted by voice call following standardized guidelines. The students in the reception group obtained informed consent from participants and they collected basic information. Subsequently, Korean Medicine doctors assessed COVID-19–related symptoms and prescribed the appropriate herbal medicine according to the KM telemedicine guidelines. The data of patients who completed the program by June 30, 2020, were analyzed.

**Results:**

From March 9 to June 30, 2020, 2324 patients participated in and completed the KM telemedicine program. Kyung-Ok-Ko (n=2285) was the most prescribed herbal medicine, and Qingfei Paidu decoction (I and II, n=2053) was the second most prescribed. All COVID-19–related symptoms (headache, chills, sputum, dry cough, sore throat, fatigue, muscle pain, rhinorrhea, nasal congestion, dyspnea, chest tightness, diarrhea, and loss of appetite) improved after treatment (*P*<.001).

**Conclusions:**

The KM telemedicine center has provided medical service to 10.8% of all patients with COVID-19 in South Korea (as of June 30, 2020), and it is still in operation. We hope that this study will help to establish a better health care system to overcome COVID-19.

## Introduction

Declared a pandemic in early 2020, COVID-19 has spread beyond China, reaching all around the world. The World Health Organization (WHO) increased its assessment of the risk of spread and impact of COVID-19 to “very high” at the global level on February 28 [1]. On November 15, 2020, the number of confirmed cases of COVID-19 exceeded 53.7 million, and the death toll was approximately 1.3 million [2]. In South Korea, since the day the first patient was confirmed on January 20, 2020, the total number of confirmed cases on September 1 exceeded 20,000. There was an outbreak of COVID-19 in Daegu and Gyeongbuk (Daegu-Gyeongbuk) due to religious gatherings attended by many people; consequently, the total number of cases rapidly increased from 30 on February 17 to 8086 on March 14. This health care surge led to a shortage of hospital beds, medical institutions, and medical personnel, as well as a gap in the management of patients.

**Figure 1:**
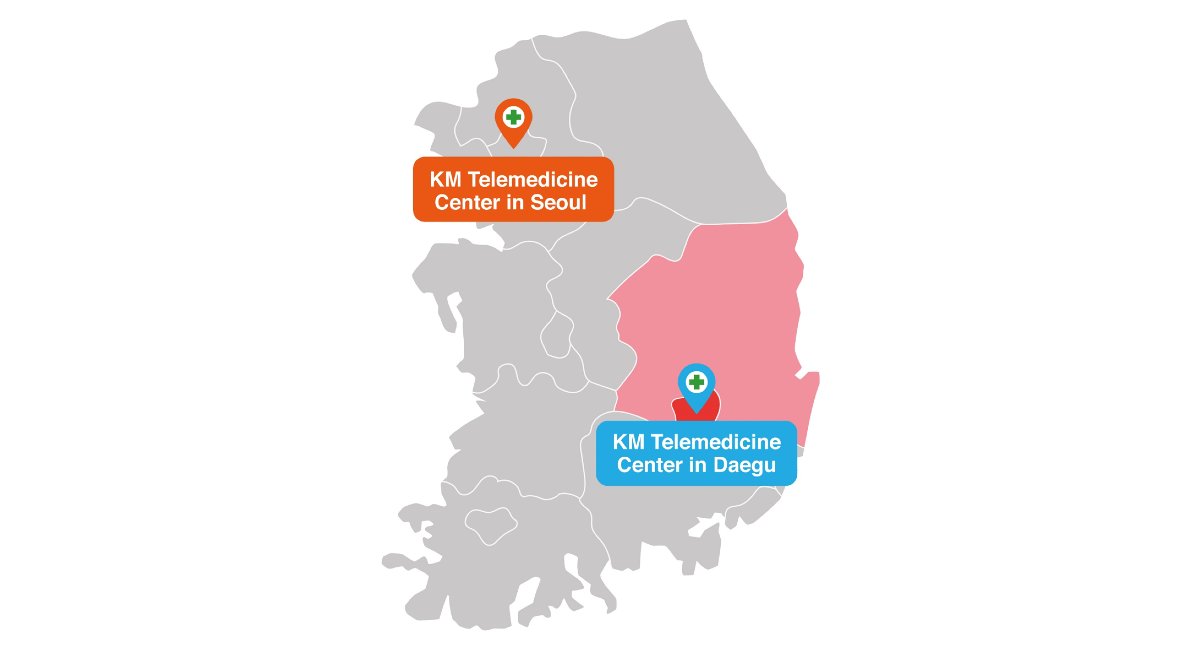
Locations of the COVID-19 telemedicine centers of Korean Medicine. KM: Korean Medicine.

**Figure 2:**
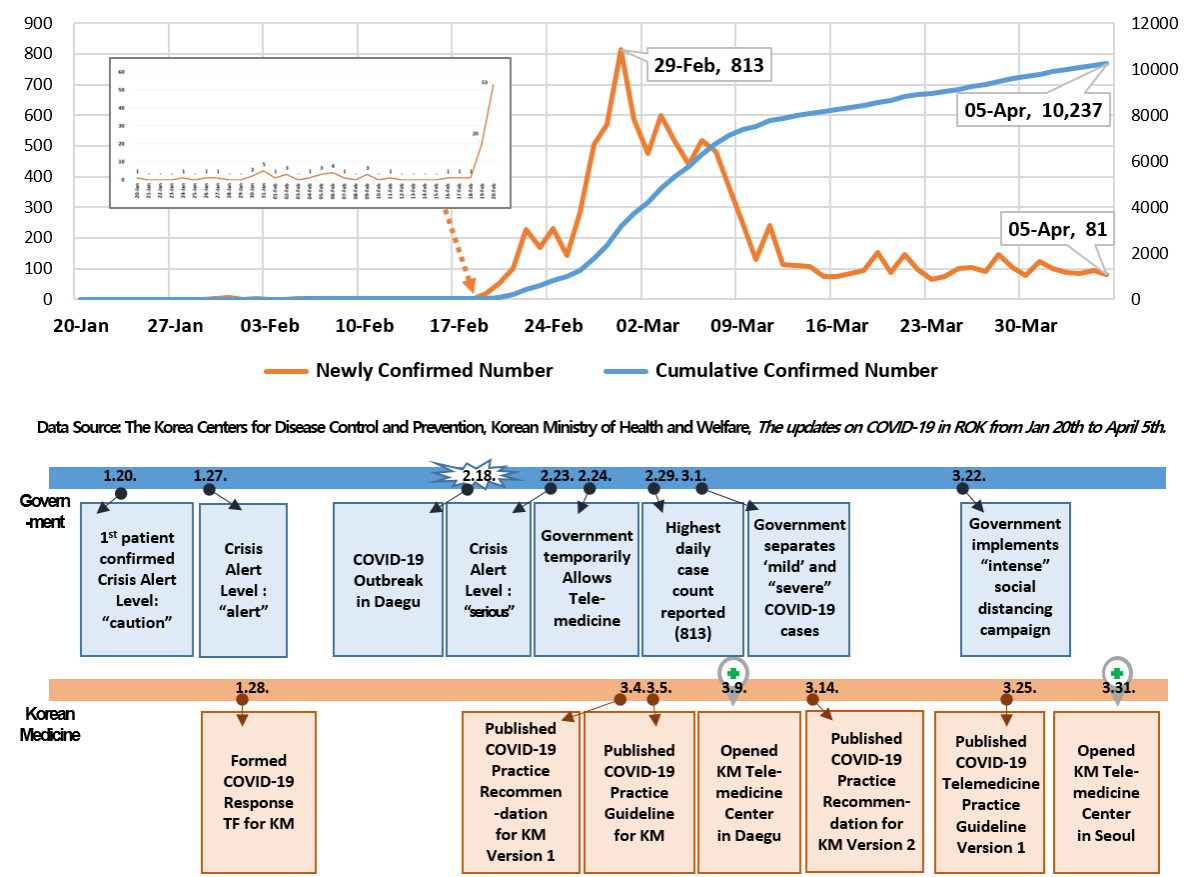
COVID-19 confirmed cases in South Korea over time. KM: Korean Medicine; ROK: Republic of Korea; TF: task force.

The Association of Korean Medicine (AKOM) created a COVID-19 telemedicine center of Korean Medicine (KM telemedicine center) at the Daegu Korean Medicine Hospital to provide medical services via telephone to marginalized patients, on March 9, 2020. After the outbreak in Daegu-Gyeongbuk was stabilized, AKOM also established an additional telemedicine center in Seoul (Figures 1 and 2). Telemedicine, in principal, is prohibited in South Korea; however, the government temporarily allowed telephone counseling or prescriptions due to COVID-19. At the KM telemedicine center, herbal medicines were provided to patients with suspected and confirmed COVID-19 for the management of COVID-19–related symptoms following the guideline on COVID-19 telemedicine service of Korean Medicine (KM telemedicine guideline). The KM telemedicine guideline was based on three previously published guidelines in South Korea and China, “Recommendations on COVID-19 Korean Medicine (AKOM)” [3], “Clinical Guidelines on COVID-19 Korean Medicine” (the KM Professor Council of Internal Medicine of the Respiratory System) [4], and the Chinese government guidelines titled “Notice on the Issuance of the New 7th Version of the COVID-19 Diagnosis and Treatment Guidelines” [5].

It has been reported that herbal medicines have been effective in reducing COVID-19 symptoms [6,7] in patients with mild symptoms and may be an alternative method to prevent the worsening of COVID-19 symptoms [8]. In particular, Qingfei Paidu decoction, which has been used for fever in Traditional Chinese Medicine (TCM), is strongly recommended for patients with confirmed COVID-19 [5,9]. This is the first study to report clinical cases of using herbal medicines for the treatment of COVID-19 in South Korea. This study aimed to describe patient symptoms and the use of medication provided by the KM telemedicine center and examine the clinical possibility of herbal medicines for COVID-19.

## Methods

### KM Telemedicine Center Operations

The KM telemedicine center consisted of an operation group, reception group, medical group, and advisory group. The operation group included AKOM executives and staff who managed the overall operation of the telephone medical center, including financial arrangements, the publicization of the KM telemedicine program, the recruitment of volunteers, and the delivery of herbal medicines. The reception group consisted of students from colleges of Korean Medicine; the medical group included Korean Medicine doctors (KMDs). Both groups were recruited voluntarily. KMDs who worked at the telemedicine center were educated in advance on how to make diagnoses, prescribe medicines, and write electronic charts. The advisory group consisted of professors from colleges of Korean Medicine, experts from academic societies, and experienced clinicians. In addition, several herbal pharmaceutical companies provided herbal medicines to the KM telemedicine center, and the rest of the expenditures, including delivery, meals, and electronic devices, were fundraised by KMDs.

### KM Telemedicine Center Procedures

All KM telemedicine procedures are conducted by voice call following standardized guidelines. Patients who want to receive KM telemedicine service voluntarily call the representative number +82-1688-1075. The students in the reception group explain the overall process and obtain the informed consent of those participating in the KM telemedicine program. They collect basic information, including the patient’s name, sex, age, address, smoking habits, date of positive COVID-19 test, infection route, and quarantine start/end date. They then schedule a telephone consultation with a KMD. KMDs then call the patients to investigate their medical history, medications, and underlying diseases, and instruct them to measure their body temperature and pulse rate. The KMDs assess COVID-19–related symptoms including pyrexia, rhinorrhea, headache, sputum, sore throat, and diarrhea, and consider pattern identification of Korean Medicine theory. They then prescribe the appropriate herbal medicine according to those presented in the KM telemedicine guidelines.

**Figure 3:**
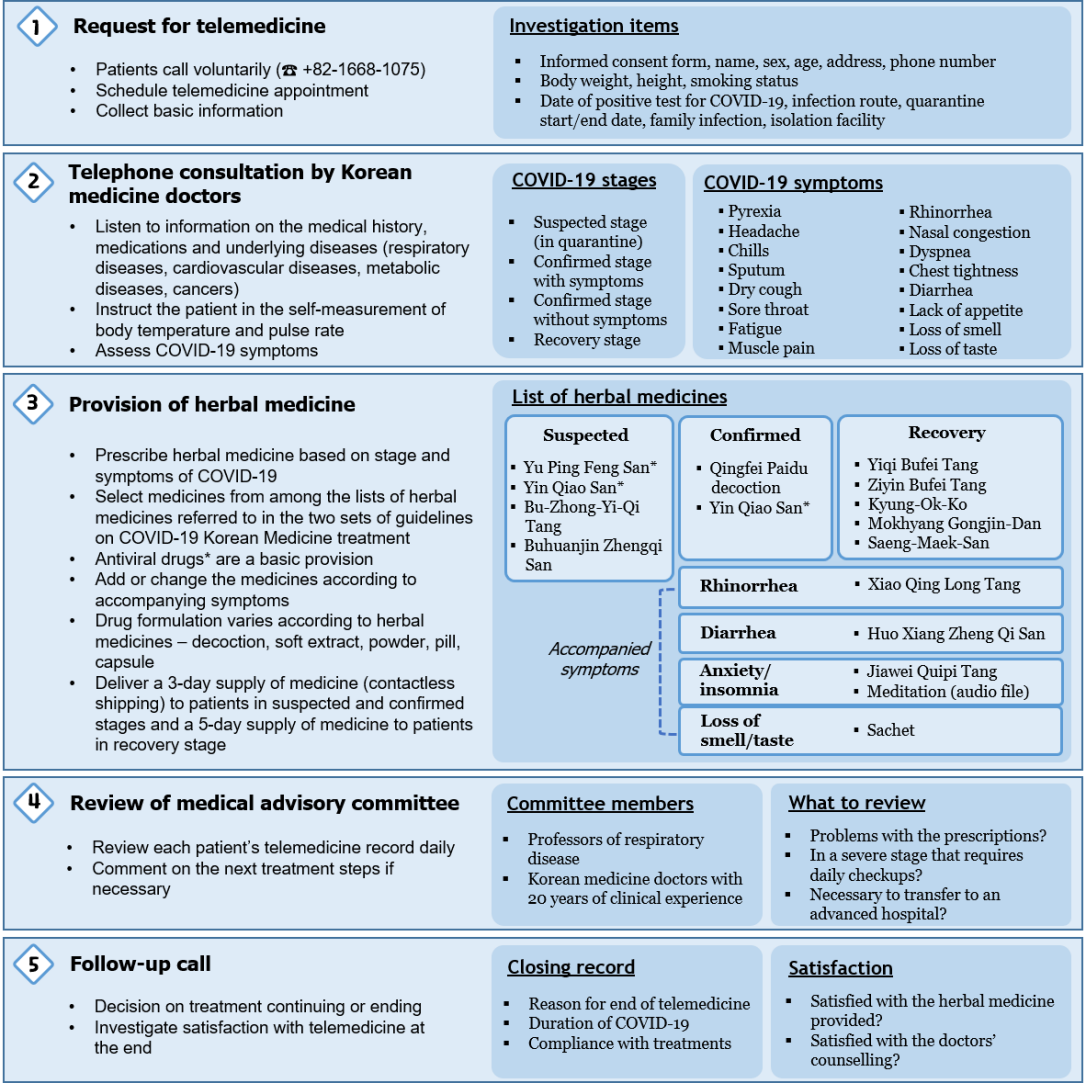
Flow diagram of steps undertaken by the COVID-19 telemedicine center of Korean Medicine.

According to the guidelines, patients with COVID-19 were first classified as uncertain suspected patients, confirmed patients (mild), asymptomatic patients, and recovery patients. When the KM telemedicine center first began operating, confirmed patients who were not hospitalized because of mild symptoms and suspected patients who had been in close contact with patients with confirmed COVID-19 were targeted by the program. As the number of cured people has gradually increased, recovering patients who are within 14 days of having tested negative for COVID-19 have been included. After the first telephone consultation, follow-up telemedicine consultations take place every 4-5 days until patients are fully recovered. The suspected patients and confirmed patients are provided with a 3-day supply of herbal medicine by contactless shipping, and recovering patients receive a 5-day supply of herbal medicine. The advisory group reviews every medical record and identifies critical patients who need intensive care, recommending transfer to advanced hospitals if necessary. In addition, the advisory group may intervene during telephone consultation in real time to discuss treatment directions with the medical team. When a patient has recovered from COVID-19 and has no other symptoms, the telemedicine ends following a decision by the KMDs (Figure 3).

### KM Telemedicine Center Interventions

For patients who have tested positive for COVID-19, the basic herbal medicine provided at the KM telemedicine center is Qingfei Baidu decoction. There are two kinds of Qingfei Baidu decoction: I is the original, and its components are the same as the Chinese Qingfei Baidu decoction, while II is the same as I but without Ephedra Herba. Qingfei Baidu decoction (II) is prescribed to those with high potential for palpitation, a side effect of Ephedra Herba. Yu Ping Feng San and Yin Qiao San have antiviral effects; therefore, they are provided to patients with suspected COVID-19. Yin Qiao San (instead of Qingfei Baidu decoction) can be considered for confirmed patients with sore throats and headaches.

Since many patients with COVID-19 complain of digestive symptoms such as diarrhea and lack of appetite, Huo Xiang Zheng Qi San, Bu-Zhong-Yi-Qi-Tang, and Buhuanjin Zhengqi San are included in the herbal medicine list for the treatment of COVID-19. KM doctors may consider other herbal medicines according to patients’ accompanying symptoms, such as Xiao Qing Long Tang for rhinorrhea and Jiawei Quipi Tang for anxiety. In particular, even if the respiratory symptoms are mild, many cases of emotional problems have been reported due to isolation or anxiety about the disease’s exacerbation. It has been reported that quarantined patients with COVID-19 suffer from insomnia, anxiety, anger, overthinking, decreased concentration, and a loss of energy [10]. Emotional symptoms such as insomnia and anxiety can continue after quarantine release; therefore, Jiawei Quipi Tang and a meditation audio file are provided in those cases. If patients have lost their sense of smell or taste, a sachet consisting of 7 herbs is delivered along with the herbal medicine. In the KM telemedicine program, managing recovering patients is important because many patients still complain about physical or emotional problems even after testing negative for COVID-19. We found that Kyung-Ok-Ko is the second most frequently prescribed medicine. Yiqi Bufei Tang is for patients with digestive symptoms, and Ziyin Bufei Tang is for patients with respiratory symptoms. Saeng-Maek San targets those who have dry mouth and sweating. Kyung-Ok-Ko and Mokhyang Gongjin-Dan are used to enhance stamina after the onset of viral infection. The formulation of the herbal medicines has varied because they were donated by herbal pharmaceutical companies and KMDs (Multimedia Appendix 1).

### Statistical Analyses

A statistician performed the statistical analyses for intention to treat (ITT) using SAS software (Version 9.4, SAS Institute). Data of patients who completed the program by June 30 were analyzed. Continuous variables (patient satisfaction) were displayed as mean (SD), median, and interquartile range. Categorical variables (sex, age, region, diagnosis, number of calls, residence of patients, underlying disease, prescriptions, and COVID-19 symptom severity) were shown as frequencies. The Wilcoxon signed-rank test was used for 13 COVID-19 symptoms to examine significant differences before and after treatment.

### Ethics Approval

This study could not receive original individual medical charts. However, we did receive the medical activities data reported by AKOM. An institutional review board at the Korea Institute of Oriental Medicine permitted this study’s use of medical records (Number I-2006/005-001-02).

## Results

### Basic Information About the KM Telemedicine Center’s Operation and Patient Characteristics

The KM telemedicine center for COVID-19 was set up in Daegu on March 9, 2020, to provide medical services, and another center was opened in Seoul on March 31. By June 30, the total number of patients was 2324. The number of confirmed cases (excluding the suspected cases) was 1392, which was 10.9% of the nationwide confirmed cases of COVID-19 as of June 30. The number of participating KMDs and students was 546 and 332, respectively. Detailed information about patients is described in Table 1.

**Table 1:** Characteristics of patients enrolled in the Korean Medicine telemedicine program for COVID-19 (N=2324).

Characteristics			Patients, n (%)
**Sex**			
	Male		569 (24.5)
	Female		1755 (75.5)
**Age (years)**			
	0-19		97 (4.2)
	20-39		555 (23.9)
	40-59		1121 (48.2)
	≥60		550 (23.7)
	Unknown		1 (0.0)
**Region**			
	Daegu and Gyeongbuk		2196 (94.5)
	Seoul		32 (1.4)
	Other		94 (4.1)
**Diagnosis (based on first consultation)**			
	Suspected cases		45 (1.9)
	Confirmed cases		211 (9.1)
	Recovering cases after discharge		2009 (86.4)
	Other, unknown		59 (2.6)
**Number of calls (per patient)**			
	1		166 (7.1)
	2		329 (14.2)
	3		432 (18.6)
	4		338 (14.5)
	≥5		1059 (45.6)
**Residence of patients (based on first consultation)**			
	Home		2165 (93.2)
	Facility		69 (2.9)
	Hospital		43 (1.9)
	Other, unknown		47 (2.0)
**Underlying diseases**			
	**Hypertension (n=1912)**		
		No	1548 (81.0)
		Yes, not taking medication	44 (2.3)
		Yes, taking medication	320 (16.7)
	**Diabetes mellitus (n=1859)**		
		No	1690 (90.9)
		Yes, not taking medication	25 (1.3)
		Yes, taking medication	144 (7.7)
	**Hyperlipidemia (n=1844)**		
		No	1626 (88.2)
		Yes, not taking medication	72 (3.9)
		Yes, taking medication	146 (7.9)
	**Cancer (n=1734)**		
		No	1661 (95.8)
		Yes, not taking medication	49 (2.8)
		Yes, taking medication	24 (1.4)
	**Chronic respiratory diseases (n=1726)**		
		No	1611 (93.3)
		Yes, not taking medication	82 (4.8)
		Yes, taking medication	33 (1.9)

### Results of the KM Telemedicine Center’s Operation

Kyung-Ok-Ko (n=2285) was the most prescribed herbal medicine, with Qingfei Paidu decoction (I and II, n=2053) following as the second most prescribed medicine. Ziyin Bufei Tang (n=1780) and Yiqi Bufei Tang (n=1499), which are herbal medicines for the recovery stage, were the third and fourth most prescribed medicines (Table 2).

**Table 2:** Frequency of prescriptions by the Korean Medicine telemedicine program for COVID-19.

Prescription	Uses	First call, n (%)	Others, n (%)	Total, n (%)
Qingfei Paidu decoction I	Exogenous fever and influenza	448 (13.4)	758 (6.9)	1206 (8.4)
Kyung-Ok-Ko	Fatigue, weak condition, and dry cough	439 (13.2)	1846 (16.7)	2285 (15.9)
Ziyin Bufei Tang	Short breath, fatigue, decreased appetite, dry mouth, and dry cough	369 (11.1)	1411 (12.8)	1780 (12.4)
Yiqi Bufei Tang	Short breath, fatigue, decreased appetite, and loose stool	348 (10.4)	1151 (10.4)	1499 (10.4)
Qingfei Paidu decoction II	Exogenous fever and influenza	263 (7.9)	584 (5.3)	847 (5.9)
Jiawei Quipi Tang	Anxiety, overthinking, and insomnia	234 (7.0)	840 (7.6)	1074 (7.5)
Mokhyang Gongjin-Dan	Fatigue and weak condition	176 (5.3)	747 (6.8)	923 (6.4)
Huo Xiang Zheng Qi San	Loose stool or diarrhea, chest tightness, and lack of vigor	139 (4.2)	420 (3.8)	559 (3.9)
Saeng-Maek-San	Thirst, dry mouth, cold sweat, and weak condition	82 (2.5)	259 (2.3)	341 (2.4)
Xiao Qing Long Tang	Nasal symptoms, watery rhinorrhea, and allergic rhinitis	94 (2.8)	347 (3.1)	441 (3.1)
Yin Qiao San	Sore throats and headaches with inner heat	89 (2.7)	348 (3.2)	437 (3.0)
Yu Ping Feng San	Cold, influenza, and sweating	40 (1.2)	71 (0.6)	111 (0.8)
Buhuanjin Zhengqi San	Cold, headache, and fatigue	26 (0.8)	124 (1.1)	150 (1.0)
Other	N/A^a^	584 (17.5)	2129 (19.3)	2713 (18.9)
Total^b^	N/A	3331 (100.0)	11,035 (100.0)	14,366 (100.0)

^a^N/A: not applicable.

^b^Due to duplicate medication orders, the total number of prescriptions exceeded the number of patients.

All variables were analyzed with data from the patients who completed the KM telemedicine center’s program by June 30. The difference in clinical symptoms related to COVID-19 before and after treatment are presented in Table 3. All 13 symptoms (headache, chills, sputum, dry cough, sore throat, fatigue, muscle pain, rhinorrhea, nasal congestion, dyspnea, chest tightness, diarrhea, and loss of appetite) improved after treatment (all *P*<.001). However, due to missing data, there were large differences in the analyzed numbers between the first and last call. The patient satisfaction score for treatment was 8.3 (SD 1.78) out of 10 and the convenience of the KM telemedicine center system was rated 9.3 (SD 1.27) out of 10. Patients gave high scores when asked about their willingness to recommend the KM telemedicine center to acquaintances (9.2, SD 1.58) and use Korean Medicine treatments (9.1, SD 1.54; Table 4).

**Table 3:** Changes in COVID-19 symptoms before and after accessing the Korean Medicine telemedicine program.

COVID-19 symptoms and severity			At first call, n (%)		At last call, n (%)		P value^a^
**Headache**						<.001	
	Severe	3 (0.1)		0 (0.0)		N/A^b^	
	Moderate	49 (2.4)		3 (0.2)		N/A	
	Mild	361 (17.4)		112 (9.3)		N/A	
	None	1658 (80.1)		1092 (90.5)		N/A	
	Total	2071 (100.0)		1207 (100.0)		N/A	
**Chills**						<.001	
	Severe	2 (0.1)		0 (0.0)		N/A	
	Moderate	11 (0.5)		0 (0.0)		N/A	
	Mild	207 (10.0)		57 (4.7)		N/A	
	None	1843 (89.3)		1158 (95.3)		N/A	
	Total	2063 (100.0)		1215 (100.0)		N/A	
**Sputum**						<.001	
	Severe	12 (0.6)		2 (0.2)		N/A	
	Moderate	43 (2.0)		10 (0.8)		N/A	
	Mild	707 (33.4)		308 (24.0)		N/A	
	None	1357 (64.0)		963 (75.1)		N/A	
	Total	2119 (100.0)		1283 (100.0)		N/A	
**Dry cough**						<.001	
	Severe	3 (0.1)		0 (0.0)		N/A	
	Moderate	116 (5.5)		16 (1.3)		N/A	
	Mild	640 (30.6)		233 (18.7)		N/A	
	None	1335 (63.8)		994 (80.0)		N/A	
	Total	2094 (100.0)		1243 (100.0)		N/A	
**Sore throat**						<.001	
	Severe	2 (0.1)		0 (0.0)		N/A	
	Moderate	43 (2.1)		2 (0.2)		N/A	
	Mild	355 (17.0)		117 (9.5)		N/A	
	None	1684 (80.8)		1113 (90.3)		N/A	
	Total	2084 (100.0)		1232 (100.0)		N/A	
**Fatigue**						<.001	
	Severe	15 (0.7)		3 (0.2)		N/A	
	Moderate	164 (8.1)		27 (2.2)		N/A	
	Mild	616 (30.5)		325 (26.7)		N/A	
	None	1223 (60.6)		860 (70.8)		N/A	
	Total	2018 (100.0)		1215 (100.0)		N/A	
**Muscle pain**						<.001	
	Severe	7 (0.3)		0 (0.0)		N/A	
	Moderate	60 (2.9)		8 (0.7)		N/A	
	Mild	334 (16.2)		108 (9.1)		N/A	
	None	1661 (80.6)		1066 (90.2)		N/A	
	Total	2062 (100.0)		1182 (100.0)		N/A	
**Rhinorrhea**						<.001	
	Severe	9 (0.4)		0 (0.0)		N/A	
	Moderate	43 (2.1)		4 (0.3)		N/A	
	Mild	316 (15.3)		164 (13.6)		N/A	
	None	1694 (82.2)		1042 (86.1)		N/A	
	Total	2062 (100.0)		1210 (100.0)		N/A	
**Nasal congestion**						<.001	
	Severe	5 (0.2)		1 (0.1)		N/A	
	Moderate	42 (2.0)		4 (0.3)		N/A	
	Mild	386 (18.7)		118 (9.8)		N/A	
	None	1627 (79.0)		1078 (89.8)		N/A	
	Total	2060 (100.0)		1201 (100.0)		N/A	
**Dyspnea**						<.001	
	Severe	17 (0.9)		0 (0.0)		N/A	
	Moderate	36 (1.9)		8 (0.7)		N/A	
	Mild	413 (21.7)		137 (12.8)		N/A	
	None	1439 (75.5)		924 (86.4)		N/A	
	Total	1905 (100.0)		1069 (100.0)		N/A	
**Chest tightness**						<.001	
	Severe	0 (0.0)		0 (0.0)		N/A	
	Moderate	51 (2.5)		4 (0.3)		N/A	
	Mild	142 (7.0)		29 (2.5)		N/A	
	None	1844 (90.5)		1144 (97.2)		N/A	
	Total	2037 (100.0)		1171 (100.0)		N/A	
**Diarrhea**						<.001	
	Severe	6 (0.3)		1 (0.1)		N/A	
	Moderate	13 (0.6)		0 (0.0)		N/A	
	Mild	181 (8.8)		87 (7.4)		N/A	
	None	1853 (90.3)		1095 (92.6)		N/A	
	Total	2053 (100.0)		1183 (100.0)		N/A	
**Loss of appetite**						<.001	
	Severe	10 (0.5)		2 (0.2)		N/A	
	Moderate	134 (6.7)		25 (2.1)		N/A	
	Mild	449 (22.5)		154 (12.9)		N/A	
	None	1407 (70.4)		1009 (84.8)		N/A	
	Total	2000 (100.0)		1190 (100.0)		N/A	

^a^The Wilcoxon signed-rank test was performed.

^b^N/A: not applicable.

**Table 4:** Patient satisfaction with Korean Medicine telemedicine program for COVID-19.

Category	Patients, n	Mean score (SD) out of 10	Median score (IQR) out of 10
Satisfaction in terms of treatment	1570	8.3 (1.78)	8.0 (7.0-10.0)
Convenience of KM telemedicine center system	1568	9.3 (1.27)	10.0 (9.0-10.0)
Satisfaction with using telephone	1570	8.7 (1.59)	9.0 (8.0-10.0)
Willingness to recommend to acquaintances	1570	9.2 (1.58)	10.0 (9.0-10.0)
Willingness to use Korean Medicine treatments	1569	9.1 (1.54)	10.0 (8.0-10.0)

## Discussion

### Principal Findings

This study introduced the KM telemedicine center, which used Korean Medicine treatments for COVID-19 and reported the clinical symptoms of patients with COVID-19 before and after treatment. Among the confirmed cases enrolled in the KM telemedicine center, 75.5% (n=1755) Table 1) were women, while 60% of the total cumulative patients with confirmed COVID-19 in South Korea were women [11]. This difference may be because COVID-19 spread rapidly through certain religious groups that had a higher proportion of women. In addition, women's preferences regarding health care services and Korean Medicine treatment may have had an impact. The majority of patients that visited the KM telemedicine center were in Daegu-Gyeongbuk because the number of patients in that region was high until June 2020. Patients with mild illness could not be hospitalized and were isolated in their homes or other facilities; therefore, the demand for telemedicine increased.

Of the total 2324 cases, Qingfei Paidu decoction, Yiqi Bufei Tang, Ziyin Bufei Tang, and Kyung-Ok-Ko were most frequently provided to patients. As 86.4% (n=2009) of patients were in the recovery stage, Kyung-Ok-Ko, Yiqi Bufei Tang, and Ziyin Bufei Tang may be prescribed in large proportions (Figure 3). It has recently been elucidated that Qingfei Paidu decoction, which is the most recommended herbal medicine for treating COVID-19, regulates cytokine storms during the viral infection [12,13]. In addition, a clinical study in China of Qingfei Paidu decoction in addition to Western medicine was recently published [14]. This study showed that in addition to respiratory symptoms, protocols of herbal medicines have been prepared for digestive symptoms, muscle pain, and loss of taste, which are all known clinical symptoms of COVID-19. Although the results showed a statistical difference before and after treatment, it is hard to guarantee the effectiveness of herbal medicines for COVID-19 symptoms because many patients did not have symptoms at baseline. Any patient currently positive for, recovering from, or suspected of having COVID-19 could be enrolled in this study, whether symptoms were present or not. For that reason, there were many patients that responded “No” to each symptom, but many others noted having more than one symptom. It is significant that patient satisfaction with the KM telemedicine center was high. This may be because the center treated patients’ symptoms and provided emotional support through counselling.

The average score for patient satisfaction at the KM telemedicine center was 8.3. The reason the score was high may be because the center supported the treatment of patients with acute symptoms. In South Korea, there were a large number of confirmed patients in Daegu-Gyeongbuk, such that the health system’s capacity was exceeded. Patients with mild COVID-19 cases were not provided with medical management, and 5 self-quarantined patients in South Korea died at home [15]. The KM telemedicine center was established to fill the gap in health care that arises from these health care surges for patients with mild cases. However, patients gave lower scores for treatment satisfaction at the KM telemedicine center, compared with the results of other satisfaction score criteria. Although patients indicated it was convenient to use the KM telemedicine center (9.3), their satisfaction with telemedicine treatment (8.7) and satisfaction in terms of treatment were relatively low (8.3). This is because non–face-to-face treatments such as telephone treatment are limited compared to face-to-face treatment, which may lead to less satisfaction from patients. This shows that there are many challenges to solve in telemedicine, such as patient access to technology, the possibility of missing test results, and the lack of technology to replace medical devices [16,17].

There are several limitations in this study. First, only patient-reported outcomes were evaluated at the KM telemedicine center. Temperature and pulse rate were also self-measured; however, these could not be reported because of missing data and errors. In addition, there were a lot of missing data points regarding each symptom because this telemedicine center had not been designed for a study and this research is based on retrospective chart review. Second, this study showed the results of the entire program, so it could not show the effect of individual herbal medicines. Since the prescription was changed according to the clinical situation of the patients, we could not compare clinical symptoms before and after each herbal medicine was administered. Third, it was difficult to coordinate care with conventional treatment methods to manage patients with underlying diseases because the KM telemedicine center did not officially belong to the national quarantine system. Fourth, it was not possible to determine the color and shape of the tongue or face color over the telephone, all of which are meaningful in Korean Medicine theory.

Nonetheless, the KM telemedicine center attempted to test the weaknesses of telemedicine and improve the quality of medical care offered via telemedicine. It has developed guidelines to help medical staff perform standardized health care. The second edition of the guideline was published on April 1, 2020, and was complemented with feedback from the medical and advisory groups, containing considerations from real telemedicine cases. Above all, Korean Medicine is traditionally compatible with telemedicine because it collects information for diagnosis by listening to the patient's overall symptoms. In addition, an advisory committee composed of professors and experienced clinicians reviewed all patients' medical charts to verify telephone consultations of KMDs. These may have led people to feel more satisfied with the KM telemedicine center system despite the limitations of non–face-to-face treatment.

### Conclusion

This study described the background and operational result of the KM telemedicine center for COVID-19 in South Korea. The KM telemedicine center attempted to overcome the limitations of telemedicine by providing standardized guidelines and expert advice. We hope that this study will help to establish a better health care system to overcome COVID-19.

## Appendix

Multimedia Appendix 1 Composition of herbal medicines provided by the COVID-19 telemedicine center of Korean Medicine.

## Abbreviations

AKOM: The Association of Korean Medicine
KM: Korean Medicine
KMD: Korean Medicine doctor
TCM: Traditional Chinese Medicine
WHO: World Health Organization
