# Supplementary material for: Telemedicine and the Use of Korean Medicine for Patients With COVID-19 in South Korea: Observational Study
Source: JMIR Public Health Surveill. 2021 Jan 19;7(1):e20236. doi: 10.2196/20236 (PMC7817255; doi:10.2196/20236)
Supplement: Multimedia Appendix 1 [file publichealth_v7i1e20236_app1.docx]

Appendix 1. Composition of herbal medicines included in COVID-19 telemedicine of Korean medicine

| Herbal medicine | Composition (daily dosage) | Formulation |
| --- | --- | --- |
| Qingfei Paidu decoction | Ephedrae Herba 9g, Glycyrrhizae Radix Praeparata 6g, Armeniacae Semen 9g, Gypsum Fibrosum 15g, Cinnamomi Ramulus 9g, Alismatis Rhizoma 9g, Polyporus 9g, Atractylodis Macrocephalae Rhizoma 9g, Poria Sclerotium 15g, Bupleuri Radix 16g, Scutellariae Radix 6g, Pinelliae Rhizoma Praeparatum cum Zingibere et Alumine 9g, Zingiberis Rhizoma Recens 9g, Asteris Radix et Rhizoma 9g, Belamcandae Rhizoma 9g, Asari Radix 6g, Dioscoreae Rhizoma 12g, Aurantii Fructus Immaturus 6g, Citri Reticulatae Pericarpium 6g, Agastachis Herba 9g | Soft extract |
| Yin Qiao San | Lonicerae Flos 15g, Forsythiae Fructus 15g, Phragmitis Rhizoma 15g, Platycodonis Radix 9g, Menthae Herba 9g, Arctii Fructus 9g, Glycine Semen Preparata 7.5g, Glycyrrhizae Radix 7.5g, Phyllostachydis Folium 6g, Schizonepetae Spica 6g | Decoction, capsule, soft extract |
| Yu Ping Feng San | Astragali Radix Praeparata cum Melle 15g, Atractylodis Macrocephalae Rhizoma 15g  Saposhnikoviae Radix Zizyphi Fructus 7.5g | Decoction |
| Bu-Zhong-Yi-Qi-Tang | Astragali Radix 1.88g, Ginseng Radix et Rhizoma 1.25g, Atractylodis Macrocephalae Rhizoma 1.25g, Glycyrrhizae Radix 1.25g,  Angelicae Gigantis Radix 0.63g, Citri Reticulatae Pericarpium 0.63g, Bupleuri Radix 0.38g, Cimicifugae Rhizoma 0.38g | Decoction, powder |
| Buhuanjin Zhengqi San | Atractylodis Rhizoma 7.5g Magnoliae Cortex 3.75g Citri Reticulatae Pericarpium 3.75g Agastachis Herba 3.75g Pinelliae Tuber 3.75g Glycyrrhizae Radix 3.75g Zingiberis Rhizoma Recens 1.5g Zizyphi Fructus 2.01g | Soft extract |
| Huo Xiang Zheng Qi San | Agastachis Herba 12g, Perillae Folium 8g, Zingiberis Rhizoma Recens 6g, Glycyrrhizae Radix Praeparata 4g, Platycodonis Radix 4g, Arecae Pericarpium 4g, Zizyphi Fructus 4g, Pinelliae Rhizoma Praeparatum cum Zingibere et Alumine 4g, Poria Sclerotium 4g, Angelicae Dahuricae Radix 4g, Atractylodis Macrocephalae Rhizoma 4g, Citri Reticulatae Pericarpium 4g, Magnoliae Cortex 4g | Decoction |
| Xiao Qing Long Tang | Ephedrae Herba 5.64g Paeoniae Radix 5.64g  Pinelliae Tuber 5.64g Schisandrae Fructus 5.64g Cinnamomi Ramulus 3.75g Asari Radix 3.75g, Glycyrrhizae Radix 3.75g Zingiberis Rhizoma 3.75g | Capsule |
| Jiawei Quipi Tang | Longan Arillus 16g Zizyphi Semen 16g, Angelicae Gigantis Radix 8g Atractylodis Macrocephalae Rhizoma 8g Poria Sclerotium 8g Codonopsis Radix 8g Astragali Radix 8g Bupleuri Radix 8g Gardeniae Fructus 8g, Polygalae Radix Preparata cum Glycyrrhizae Radix 4g Aucklandiae Radix 4g, Glycyrrhizae Radix 2.4g, Zingiberis Rhizoma Recens 8g,  Zizyphi Fructus 8g | Decoction |
| Yiqi Bufei Tang | Astragali Radix Praeparata cum Melle 30g, Poria Sclerotium 15g Codonopsis Radix 15g, Agastachis Herba 10g Atractylodis Macrocephalae Rhizoma 10g Citri Reticulatae Pericarpium 10g, Pinelliae Rhizoma Praeparatum cum Zingibere et Alumine 9g, Amomi Fructus 6g Glycyrrhizae Radix 6g | Decoction |
| Ziyin Bufei Tang | Adenophorae Radix 15g, Ophiopogonis Radix 15g, Astragali Radix 15g, Rehmanniae Radix 15g, Paeoniae Radix 15g, Mori Cortex 10g, Lycii Radicis Cortex 10g, Aurantii Fructus 10g, Artemisiae Annuae Herba 10g, Massa Medicata Fermentata 5g, Hordei Fructus Germinatus 5g, Crataegi Fructus 5g, Glycyrrhizae Radix 5g | Decoction |
| Kyung-Ok-Ko | Mel 24.48g, Rehmanniae Radix 21.93g, Poria Sclerotium 3.06g, Ginseng Radix et Rhizoma 1.53g | Soft extract, pill |
| Mokhyang Gongjin-Dan | Ophiopogonis Radix 7.5g Ginseng Radix et Rhizoma 3.75g Schisandrae Fructus 3.75g | Pill |
| Saeng-Maek-San | Ophiopogonis Radix 7.5g Ginseng Radix et Rhizoma 3.75g Schisandrae Fructus 3.75g | Powder |
| Sachet  (Place it in pocket or bedroom and shake it often) | Artemisiae Argyi Folium, Agastachis Herba,  Menthae Herba, Angelicae Dahuricae Radix,  Aucklandiae Radix, Atractylodis Rhizoma,  Notopterygii Rhizoma et Radix, Caryophylli Flos, Rubiae Radix, Asari Radix | Herb |
